# Case Report: Regulatory T Cell-Independent Induction of Remission in a Patient With Collagenous Colitis

**DOI:** 10.3389/fmed.2021.678268

**Published:** 2021-07-19

**Authors:** Hajime Honjo, Tomohiro Watanabe, Mizuki Tomooka, Takuya Matsubara, Masashi Kono, Ikue Sekai, Akane Hara, Masayuki Kurimoto, Keisuke Yoshikawa, Yasuhiro Masuta, Yasuo Otsuka, Ryutaro Takada, Tomoe Yoshikawa, Ken Kamata, Kosuke Minaga, Shigenaga Matsui, Masatomo Kimura, Masatoshi Kudo

**Affiliations:** ^1^Department of Gastroenterology and Hepatology, Kindai University Faculty of Medicine, Osaka, Japan; ^2^Department of Diagnostic Pathology, Kindai University Hospital, Osaka, Japan

**Keywords:** collagenous colitis, CD8^+^ T cells, regulatory T cells, Foxp3, immunohistochemistry

## Abstract

Collagenous colitis (CC), a prototypical microscopic colitis, is a chronic inflammatory disorder of the colon. The diagnosis of CC depends on the pathological examination. The colonic mucosa of patients with CC is characterized by the presence of a substantially thickened collagen band (>10μm) under the surface epithelium. In addition, intraepithelial and lamina propria lymphocytes are markedly increased in patients with CC. However, the roles played by the lymphocytes accumulating in the colonic mucosa of patients with CC are poorly defined. Recent studies indicate that T cells infiltrating the colonic mucosa of patients with CC are mainly represented by CD4^+^ T cells, CD8^+^ T cells, and forkhead box P3 (FOXP3)^+^ regulatory T cells (Tregs). Given that activation of CD4^+^/CD8^+^ T cells and FOXP3^+^ Tregs usually mediates pro-inflammatory and anti-inflammatory responses, respectively, alterations in the colonic numbers of these adaptive T cells might be related to the resolution of colitis in patients with CC. We determined alterations in the composition of colonic T cells by extensive immunohistochemical (IHC) analyses in a case of CC successfully treated with budesonide and metronidazole. Colonic lamina propria immune cells mainly comprised CD3^+^ T cells, CD4^+^ T cells, CD8^+^ T cells, CD68^+^ macrophages, and FOXP3^+^ Tregs, but not CD20^+^ B cells or myeloperoxidase (MPO)^+^ granulocytes in the active phase. During remission, the numbers of CD3^+^ T cells, CD4^+^ T cells, CD8^+^ T cells, and CD68^+^ macrophages did not change significantly in the colonic lamina propria, whereas FOXP3^+^ Tregs were markedly decreased, suggesting that induction of remission was achieved in a Treg-independent manner. Thus, our study indicates that accumulation of FOXP3^+^ Tregs in the colonic mucosa of patients with CC might be a counter-regulatory mechanism reflecting persistent inflammation and that induction of remission might be achieved without activation of Tregs.

## Introduction

Microscopic colitis is a chronic inflammatory disease of the colon that often causes watery diarrhea (1–3). Its diagnosis requires a pathological examination because the colonic mucosa of patients with microscopic colitis shows slight or no abnormalities on colonoscopy (1–3). Microscopic colitis is classified into collagenous colitis (CC) and lymphocytic colitis (1–3). Prior exposure to non-steroidal anti-inflammatory drugs (NSAIDs) and proton pump inhibitors (PPIs) can trigger the development of CC, and discontinuation of these drugs is one of the treatment options (1–3). Pathologically, the colonic mucosa of patients with CC is characterized by the presence of a substantially thickened collagen band (>10μm) under the surface epithelium and accumulation of intraepithelial and lamina propria lymphocytes (1–3).

Although the immunopathogenesis of CC is poorly understood, recent studies have highlighted various roles played by T cells, mainly represented by CD3^+^ T cells and forkhead box P3 (FOXP3)^+^ regulatory T cells (Tregs), which infiltrate the colonic mucosa of patients with CC (4–7). The effector CD4^+^ and/or CD8^+^ T cells are considered to trigger chronic inflammatory reactions, which are typical in CC, through the production of proinflammatory cytokines (7, 8). However, the roles played by colonic FOXP3^+^ Tregs have been poorly defined. Given that activation of CD4^+^/CD8^+^ T cells and FOXP3^+^ Tregs usually mediates pro-inflammatory (8) and anti-inflammatory responses (9), respectively, alterations in the colonic numbers of these adaptive T cells may affect the resolution of colitis in patients with CC. Herein, we report a case of CC that was successfully treated with budesonide and metronidazole. Our extensive immunohistochemical (IHC) analysis revealed that the numbers of CD3^+^ T cells, CD4^+^ T cells, CD8^+^ T cells, and CD68^+^ macrophages were comparable in the active and remission phases. In contrast, a marked reduction in FOXP3^+^ Tregs was observed in the colonic mucosa of this patient in the remission phase. Thus, our study suggests that induction of remission in patients with CC might be achieved in a Treg-independent manner.

## Case Description

An 81-year-old man was admitted to the Kindai University Hospital because of watery diarrhea that persisted for 5 years. The patient was initially diagnosed with irritable bowel syndrome (IBS) due to the relapsing-remitting clinical course. The patient was treated with olmesartan medoxomil and celecoxib for hypertension and osteoarthritis of the knee, respectively. He also received gliclazide and alogliptin benzoate for diabetes mellitus. For IBS, he was prescribed loperamide hydrochloride and ramosetron hydrochloride. Despite these medications, he suffered from persistent watery diarrhea.

## Diagnostic Assessment

**Figure 1:**
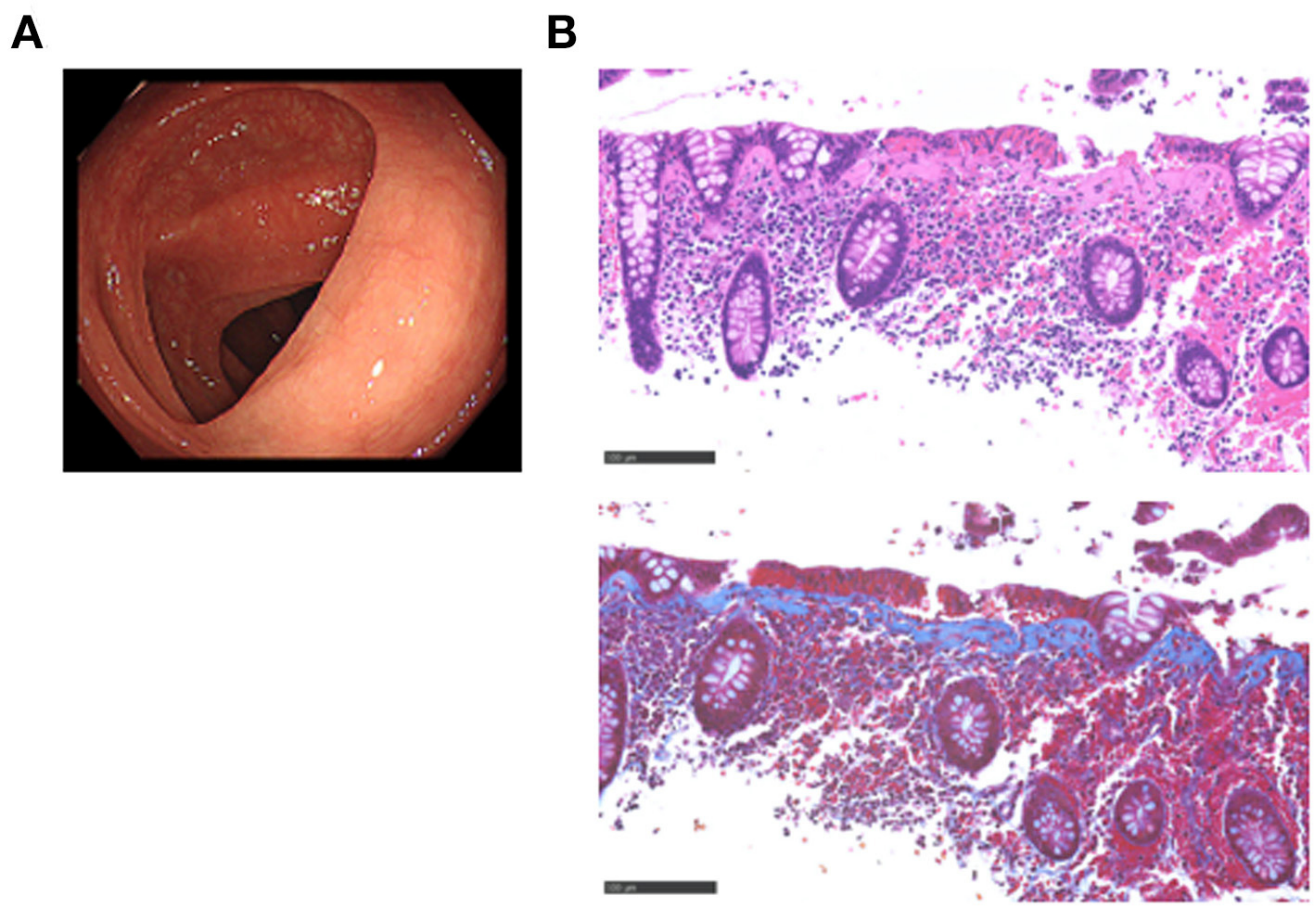
Endoscopic and pathological findings in a patient with collagenous colitis in the active phase. **(A)** Colonoscopic examination revealed vascular networks without mucosal edema in the transverse colon. **(B)** Pathological examinations of the transverse colon biopsy samples revealed the presence of a subepithelial collagen band with a thickness >10μm (Masson's trichrome staining, bottom) and massive accumulation of immune cells in the lamina propria (hematoxylin and eosin, H&E staining, top). Scale bar, 100μm. Magnification × 400.

On admission, the physical examination was unremarkable. No major abnormalities were found in blood tests, including complete blood cell counts and serum biochemistry, except for the elevated level of C-reactive protein (1.194 mg/dL; normal range, <0.14). Serum concentrations of IgG, IgM, and IgA were within the normal ranges, and the anti-nuclear antibody titer was <40. Neither pathogenic bacteria nor *Clostridium difficile* toxins were detected in the feces. Abdominal computed tomography and esophagogastroduodenoscopy performed to identify the cause of diarrhea were normal. The colonoscopic examination revealed almost normal colonic mucosa from the ascending colon to the rectum (Figure 1A). Pathological examinations using colonic biopsy samples revealed a subepithelial collagen band with a thickness >10μm in the ascending, transverse, descending, and sigmoid colon in hematoxylin & eosin staining (Figure 1B, top) and Masson's trichrome staining (Figure 1B, bottom). Massive accumulation of immune cells in the lamina propria was observed (Figure 1B). These pathological findings were consistent with CC (1, 2).

**Figure 2:**
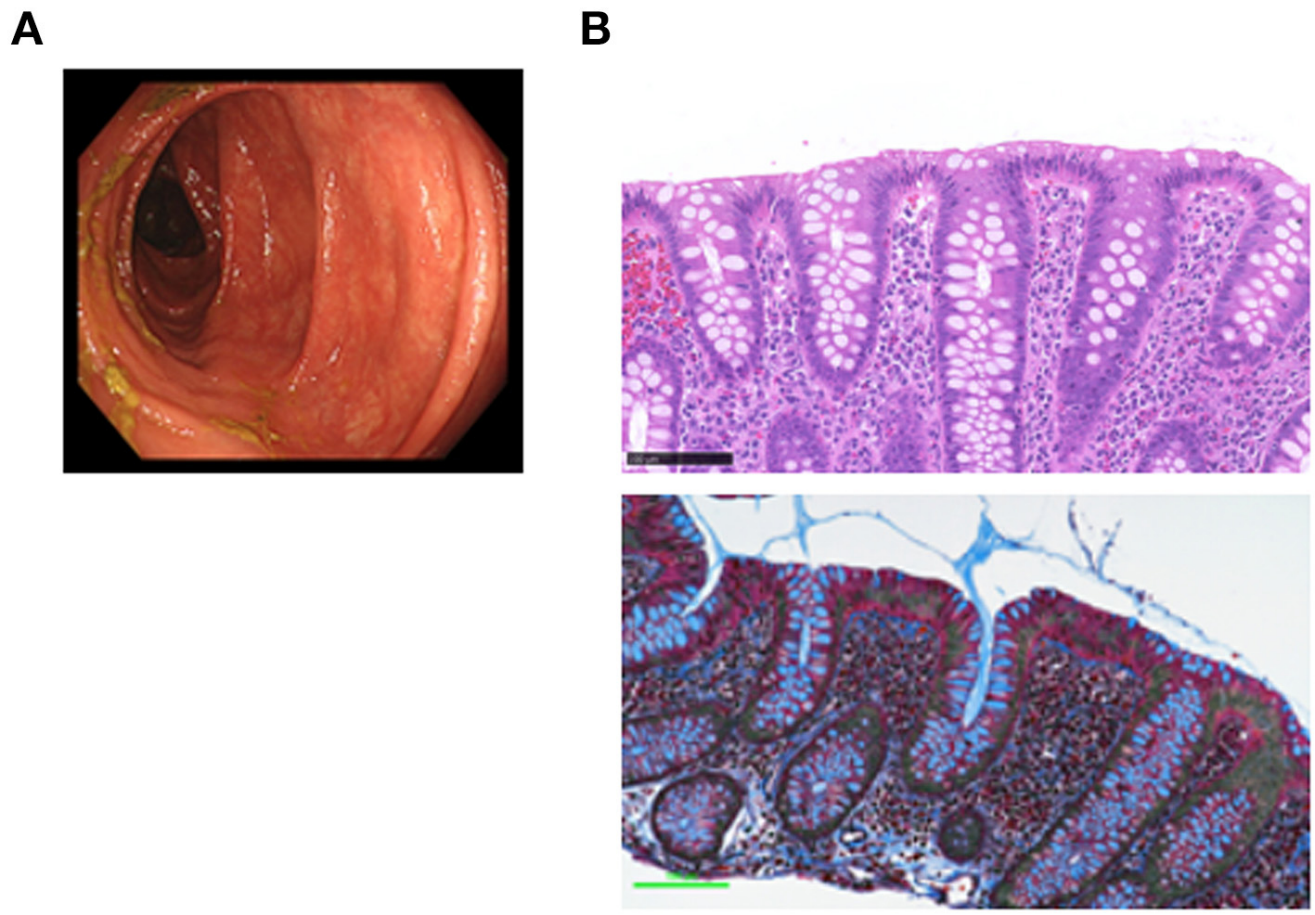
Endoscopic and pathological findings in a patient with collagenous colitis in the remission phase. **(A)** Colonoscopic examination revealed vascular networks without mucosal edema in the transverse colon. **(B)** Pathological examinations of the transverse colon biopsy samples revealed the absence of the subepithelial collagen band that was present during the active phase (Masson's trichrome staining, bottom), whereas the accumulation of immune cells in the lamina propria was still observed (hematoxylin and eosin, H&E staining, top). Scale bar, 100μm. Magnification × 400.

CC is strongly associated with the exposure to drugs (1, 2), particularly to NSAIDs and PPIs (10). Although all of such drugs were discontinued, the symptoms persisted. Administration of mesalazine and colestyramine was without effect. The patient was then treated with budesonide (9 mg/day) for 5 weeks with a tapering schedule of 3 mg every 2 weeks, according to previous studies (11). Failure of the initial 2 week treatment with budesonide alone to relieve the symptoms led us to try a combination treatment with metronidazole (1,500 mg/day) and budesonide. Watery diarrhea completely disappeared in 2 weeks after this combination therapy. Follow-up total colonoscopy performed 5 months later revealed normal colonic mucosa from the rectum to the ascending colon (Figure 2A). By that time, the subepithelial collagen band almost disappeared, whereas immune cell infiltration into the colonic mucosa was still observed (Figure 2B).

## Immunohistochemical Analysis

**Figure 3:**
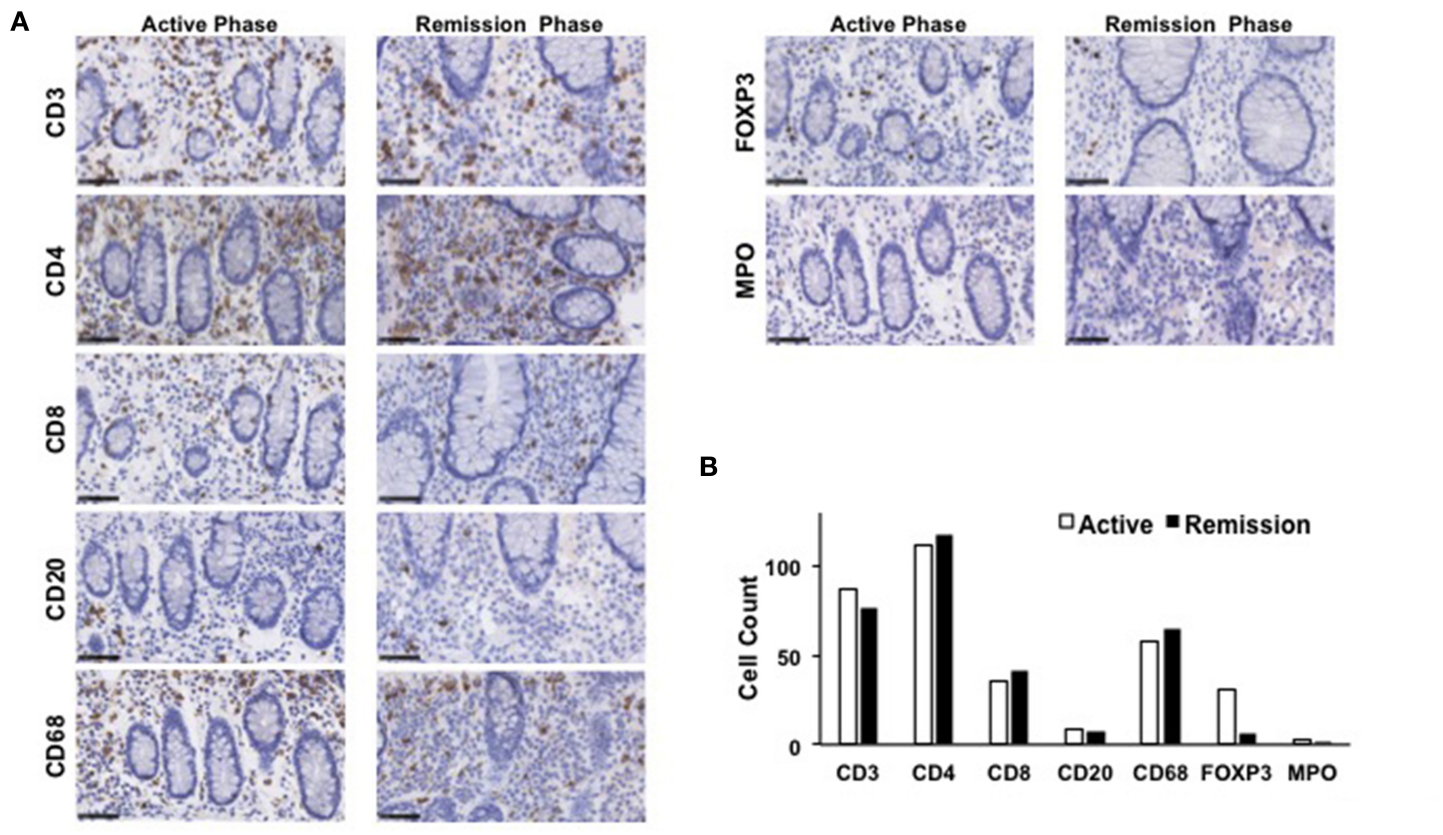
Immunohistochemical analyses of the transverse colon biopsy samples obtained from a patient with collagenous colitis during the active and remission phases. **(A)** Colonic biopsy specimens were subjected to immunohistochemical analyses to visualize CD3^+^ T cells, CD4^+^ T cells, CD8^+^ T cells, CD20^+^ B cells, forkhead box P3 (FOXP3)^+^ regulatory T cells (Tregs), CD68^+^ macrophages, and myeloperoxidase (MPO)^+^ granulocytes. Scale bar, 50μm. **(B)** Cells positive for each marker was counted. Results are expressed as means.

**Figure 4:**
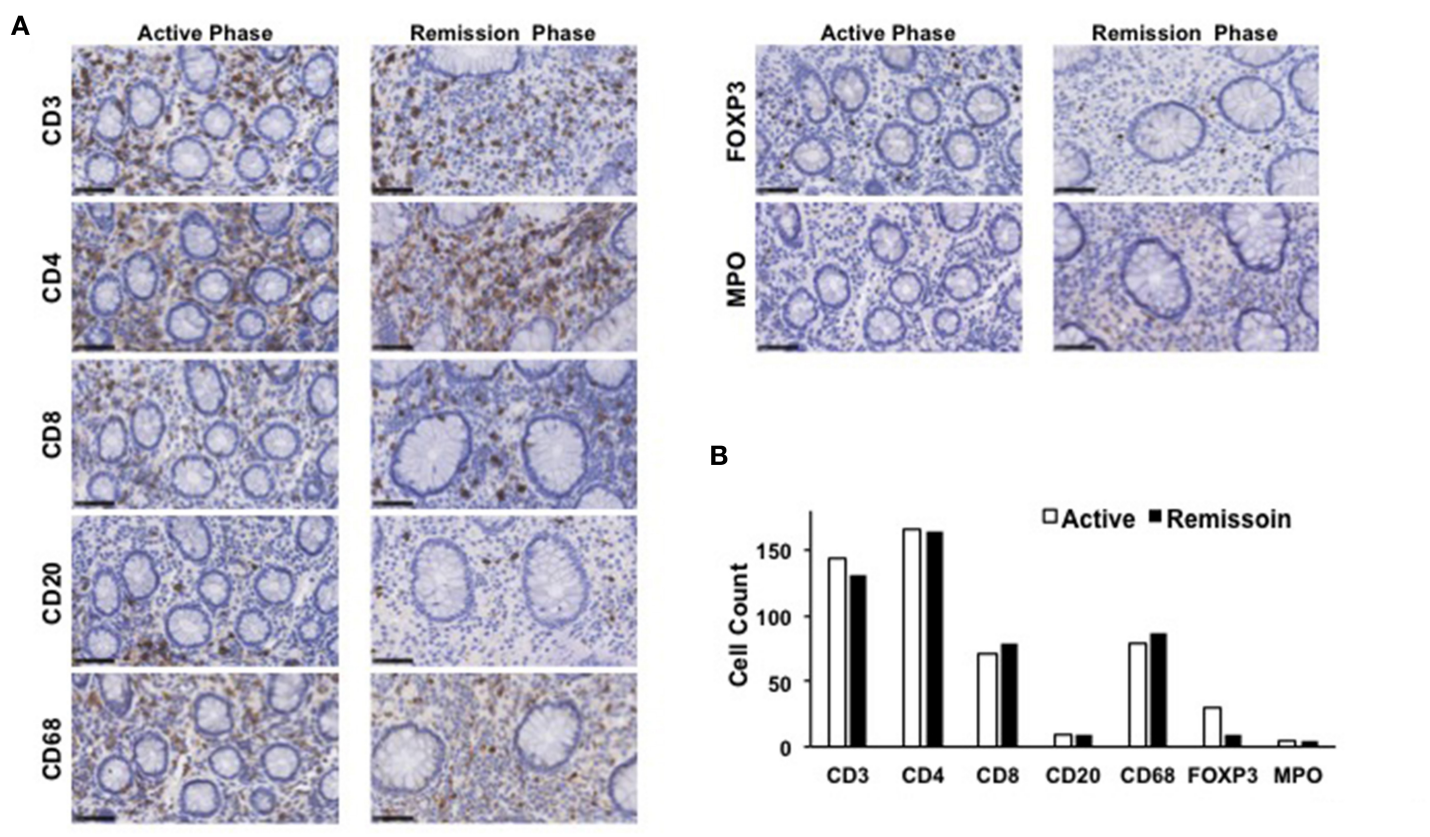
Immunohistochemical analyses of the ascending colon biopsy samples obtained from a patient with collagenous colitis during the active and remission phases. **(A)** Colonic biopsy specimens were subjected to immunohistochemical analyses to visualize CD3^+^ T cells, CD4^+^ T cells, CD8^+^ T cells, CD20^+^ B cells, forkhead box P3 (FOXP3)^+^ regulatory T cells (Tregs), CD68^+^ macrophages, and myeloperoxidase (MPO)^+^ granulocytes. Scale bar, 50μm. **(B)** Cells positive for each marker was counted. Results are expressed as means.

The mucosa in CC is characterized by the accumulation of CD4^+^ T cells, CD8^+^ T cells, and FOXP3^+^ Tregs (4, 5, 8). To ascertain the involvement of these T cell populations in the development of CC, colonic biopsy specimens were subjected to IHC analysis as previously described (12, 13). Consistent with previous reports, the mucosa of the transverse colon in this case was characterized by predominant infiltration of CD3^+^ T cells and CD68^+^ macrophages, but not of CD20^+^ B cells or myeloperoxidase (MPO)^+^ granulocytes (Figure 3). Moreover, CD3^+^ T cells that accumulated in the colonic mucosa mainly contained CD4^+^ T cells, CD8^+^ T cells, and FOXP3^+^ Tregs (Figure 3). In addition to the accumulation of adaptive T cells, the colonic mucosa of this CC case was characterized by the infiltration of CD68^+^ macrophages. The induction of remission by budesonide and metronidazole was not accompanied by significant reductions in the numbers of CD3^+^ T cells, CD4^+^ T cells, CD8^+^ T cells, or CD68^+^ macrophages in the colonic lamina propria between the active and remission phases (Figure 3). In contrast, the accumulation of FOXP3^+^ Tregs in the colonic mucosa was clearly decreased in the remission phase (Figure 3). Similar results were obtained in IHC analyses using colonic biopsy samples obtained from the ascending colon (Figure 4), descending colon, sigmoid colon and rectum (data not shown). Thus, these extensive IHC analyses strongly suggest that the induction of remission in this case of CC was accompanied by a marked reduction in FOXP3^+^ Tregs rather than of CD4^+^ or CD8^+^ effector T cells.

## Discussion

CC is a chronic inflammatory condition of the colon with poorly defined etiology that can be triggered by the exposure to NSAIDs or PPIs (1, 2). Colon usually has normal macroscopic appearance on colonoscopy in patients with CC, even in the presence of persistent diarrhea, although non-specific bowel edema is sometimes seen (1, 2). Although discontinuation of NSAIDs or PPI is often effective for patients with CC, withdrawal of NSAIDs did not improve our patient's symptoms. A combination treatment with budesonide and metronidazole, however, led to the remission of CC. We performed IHC analysis to explore the association between disease activity and the type of immune cells in the colonic mucosa. In the active disease phase, the colonic mucosa of this case was characterized by the accumulation of CD3^+^ T cells, CD4^+^ T cells, CD8^+^ T cells, FOXP3^+^ Tregs, and CD68^+^ macrophages in the colonic lamina propria. Numbers of CD3^+^ T cells, CD4^+^ T cells, CD8^+^ T cells, and CD68^+^ macrophages were not significantly different before and after treatment with budesonide and metronidazole. In contrast, induction of remission was accompanied by a marked reduction in the accumulation of FOXP3^+^ Tregs in the colonic mucosa. Thus, this case of CC is unique in that the remission was successfully induced without activation of FOXP3^+^ Tregs that normally suppress inflammatory responses.

Oral budesonide (9 mg/day) is effective and safe for the treatment of CC (11). In fact, more than 80% of patients with CC are successfully treated with budesonide (11). However, budesonide treatment alone failed to induce remission in this case. Although a combination treatment with budesonide and metronidazole led to the remission of CC, the mechanism of action of metronidazole remained unknown. Two cases with CC were successfully treated with combined treatment of metronidazole and steroid (14, 15). We speculate that alterations of the intestinal microbiota induced by metronidazole improved CC symptoms. This idea is supported by a recent study that provided evidence of the association of intestinal dysbiosis with the development of CC (16). Thus, it is possible that administration of metronidazole induces remission through alternation of intestinal microbiota composition. Therefore, alternations of intestinal microbiota composition by metronidazole may act together with anti-inflammatory effects of budesonide to induce remission in this case. However, verification of this idea requires future studies.

Our IHC analyses clearly showed accumulation of CD4^+^ T cells, CD8^+^ T cells, and FOXP3^+^ Tregs in the colonic mucosa of our patient. These data were consistent with previous studies that showed predominance of CD4^+^ T cells, CD8^+^ T cells, and FOXP3^+^ Tregs among immune cells infiltrating the colonic mucosa during CC (4–7). However, differences in the accumulation of these T cells in the colonic mucosa of patients with CC between active and remission phases have not been reported. To the best of our knowledge, this is the first case report describing alterations in pathogenic T cell populations in the colonic mucosa after the induction of remission of CC. Proinflammatory cytokines IFN-γ, IL-17, and TNF-α produced by T cells are considered to underlie the immunopathogenesis of CC (4, 7, 8). However, we could not find any differences in the numbers of colonic CD4^+^ or CD8^+^ T cells, which are major cellular proinflammatory cytokine sources. Whether proinflammatory cytokines produced by effector T cells indeed underlie the immunopathogenesis of CC requires extensive future expression analyses.

This case prompts a question regarding the role played by FOXP3^+^ Tregs in the development of CC. Tregs are a specialized effector T cell population that inhibit proinflammatory responses (9). Therefore, the paradoxical accumulation of FOXP3^+^ Tregs in the active phase is difficult to understand in terms of the pathogenesis of CC. There are two possible explanations for the relationship between the accumulation of Tregs and chronic inflammation in CC. First, the accumulation of Tregs in the colonic mucosa may be an epiphenomenon reflecting persistent inflammation associated with CC. This idea is supported by the enhanced expression of IL-10, a prototypical anti-inflammatory cytokine produced by Tregs, in patients with CC (4). Therefore, it is possible that the accumulation of Tregs in the colonic mucosa of patients with CC is a counter-regulatory mechanism. Indeed, a marked reduction in colonic FOXP3^+^ Tregs was observed in this case during the remission phase. Second, FOXP3^+^ Tregs have impaired suppressive activity against proinflammatory responses. Daferera et al. demonstrated the expansion of FOXP3^+^ T cells devoid of suppressive activity in the colonic mucosa of patients with CC (17). This idea clearly explains the persistent inflammation in patients with CC, despite the presence of FOXP3^+^ T cells. Thus, it remains unclear whether FOXP3^+^ T cells able to inhibit proinflammatory responses accumulate in the colonic mucosa of patients with CC. In either case, the results of our IHC analyses are interesting in that CC activity correlated with the degree of colonic accumulation of the FOXP3^+^ Tregs, but did not correlate with the numbers of the effector CD4^+^ or CD8^+^ T cells. It should be noted, however, that confirmation of these data obtained in IHC analyses requires quantitative analyses using flow-cytometry and polymerase chain reaction. Therefore, it is too early to consider that remission of CC occurs independent of activation of Tregs expressing FOXP3.

In conclusion, we describe a case of CC successfully treated with budesonide and metronidazole, in which active colonic lesions were infiltrated by CD3^+^ T cells, CD4^+^ T cells, CD8^+^ T cells, FOXP3^+^ Tregs, and CD68^+^ macrophages. Disease activity correlated with the degree of accumulation of FOXP3^+^ Tregs, but not with the numbers of effector CD4^+^ or CD8^+^ T cells. As the roles played by these T cells, especially FOXP3^+^ Tregs, in the immunopathogenesis of CC are poorly understood, future studies should address the molecular mechanisms of CC by focusing on the function of CD4^+^ T cells, CD8^+^ T cells, and FOXP3^+^ Tregs.

## Data Availability Statement

The original contributions presented in the study are included in the article/supplementary material, further inquiries can be directed to the corresponding author/s.

## Ethics Statement

The studies involving human participants were reviewed and approved by Kindai University Faculty of Medicine. The patients/participants provided their written informed consent to participate in this study.

## Author Contributions

HH, TW, MT, TM, MKon, IS, AH, MKur, KY, YM, YO, RT, and SM took care of the patient. HH and TW drafted the manuscript. TY, KK, KM, and MKud edited and revised the manuscript. MKim performed pathological analyses. All authors contributed to the article and approved the submitted version.

## Conflict of Interest

The authors declare that the research was conducted in the absence of any commercial or financial relationships that could be construed as a potential conflict of interest.
